# Single cell transcriptome analysis identified a unique neutrophil type associated with Alzheimer’s disease

**DOI:** 10.1186/s12979-024-00448-x

**Published:** 2024-06-25

**Authors:** Xiaolin Zhang, Guiqin He, Yixuan Hu, Boren Liu, Yuliang Xu, Xia Li, Xinyou Lv, Jin Li

**Affiliations:** 1 Shanghai Key Laboratory of Psychotic Disorders, Brain Health Institute, National Center for Mental Disorders, National Center for Mental Disorders, Shanghai Mental Health Center, Shanghai Jiaotong University School of Medicine, Shanghai, 200030 China; 2 Shanghai Institute of Materia Medica, Chinese Academy of Sciences, Shanghai, 201210 China; 3 University of Chinese Academy of Sciences, Beijing, China; 4 School of Pharmaceutical Sciences, Southern Medical University, Guangzhou, 510515 China; 5 Shanghai Mental Health Center, Shanghai Jiao Tong University School of Medicine, Shanghai, 200030 China; 6 Department of Psychology, School of Humanities and Social Sciences, University of Science and Technology of China, Hefei, 230026 Anhui China; 7 Institute of Public Health Sciences, Division of Life Sciences and Medicine, University of Science and Technology of China, Hefei, 230026 Anhui China

**Keywords:** Alzheimer’s disease, Neutrophils, Single cell RNA sequencing, CCRL2

## Abstract

**Background:**

Neutrophils play an essential role in Alzheimer’s disease (AD) pathology. However, the extent of their heterogeneity remains poorly explored, particularly in the context of developing novel therapies targeting these cells.

**Results:**

We investigate the population structure of neutrophils purified from peripheral blood samples of AD mice. Utilizing single cell RNA sequencing, we comprehensively map neutrophil populations into six distinct clusters and find that the Neu-5 subset is specially enriched in AD mice. This subset exhibits fewer specific granules and a lower mature score. Gene ontology (GO) analysis reveals that genes involved in cytokine-mediated signaling are downregulated in the Neu-5 cluster. Furthermore, we identify the *Ccrl2* gene is specifically upregulated in this subgroup, which is confirmed by flow cytometry in AD mice. Finally, immunohistochemical staining indicates that CCRL2 protein is increased in the brains of AD mice.

**Conclusions:**

We identify a unique CCRL2 positive neutrophil cluster, that is specifically enriched in the peripheral blood of AD mice.

**Supplementary Information:**

The online version contains supplementary material available at 10.1186/s12979-024-00448-x.

## Background

Alzheimer’s disease (AD) is one of the most common forms of dementia in elderly. It is characterized by a slowly-progressing neurodegenerative disorder, starting with mild cognitive impairment and gradually culminating in severe decline of memory and the ability to execute daily activities [1]. The neuropathological hallmarks of AD include aggregates of amyloid beta (Aβ) and hyperphosphorylated tau proteins, neuronal loss and synaptic dysfunction [2]. More than 35 million people suffer from AD worldwide and as no effective treatments are available currently, it imposes a heavy social and financial burdens [3, 4].

It is now well established that both the innate and adaptive immune cells are present in the brain parenchyma and meninges [5, 6]. Emerging evidence suggests that adaptive immune response, such as T cells and B cells, is closely associated with AD pathogenesis [7–9]. Several studies have reported an increase in T cells in the cerebrospinal fluid, meninges, and hippocampus in post-mortem tissue of patients with AD, as well as in both Aβ and tau mouse models [10–12]. Deletion of peripheral immune cells, including T and B lymphocytes, significantly accelerated Aβ pathology [13]. In addition, mature B cells have been observed in the brain parenchyma of AD transgenic mice and infiltration of B cells has been associated with accelerated progression of AD pathology [14, 15]. Another study found B lymphocytes could mitigate Aβ pathology and memory impairments in a transgenic AD mouse model [16].

However, information about the role of innate immune system especially focusing on neutrophils in AD pathology remains limited. Clinical studies show that CD11b integrin and reactive oxygen species from blood neutrophils are increased in AD patients [17, 18]. In AD mice, Zenaro et al. observed neutrophils accumulate in brain areas with Aβ deposits and produce neutrophil extracellular traps, promoting cognitive decline [19]. Another report suggested neutrophils adhere in brain capillary segments and block blood flow, leading to memory impairment [20]. Therefore, neutrophils in blood indeed play a critical role in AD pathology. Yet, different neutrophil populations adapt to and differ depending on the microenvironment [21–23]. In AD pathology, which subpopulations are involved and the underlying molecular mechanism remains unknown.

In this study, we adopt single-cell RNA sequencing (scRNA-seq) to explore the neutrophil heterogeneity in AD mouse model. Following an unsupervised analysis, we identified six neutrophil subpopulations with distinct signature genes, of which the Neu-5 and Neu-6 clusters were significantly increased in AD mice. By characterization, the Neu-5 showed the less specific granule while the highest immature score and chemokines. Finally, flow cytometric analysis and immunohistochemistry confirmed the Neu-5 cluster was upregulated and infiltrated into the brain. Overall, we mapped the neutrophils in blood of AD mice and identified a specific cluster associated with AD progression.

## Methods

### Mice

All experimental mice, including 7-month-old female APP/PS1 transgenic mice, 5×FAD transgenic mice and C57BL/6J mice, were purchased from SPF (Beijing) Biotechnology Co., Ltd. The mice were housed under a standard 12-h dark–light cycle in a temperature-controlled environment (22–25 °C with 40–60% humidity) with food and water provided ad libitum. All animal procedures were approved by the Institutional Animal Care and Use Committees of Shanghai Mental Health Center.

### Neutrophil isolation

To isolate neutrophils from peripheral blood, 7-month-old female APP/PS1 transgenic mice were anesthetized with isoflurane. Whole blood was collected via cardiac puncture in EDTA-coated tubes. One part blood was mixed with nine parts Ammonium Chloride Solution (STEMCELL, 07800) and laid on ice for 15 min. After centrifugation (300 g, 6 min, 4 °C), cell pellets were washed once with PBS containing 2% fetal bovine serum (FBS) and 1 mM EDTA. Cell pellet was resuspended at 1 × 10^8^ nucleated cells/mL in PBS containing 2% FBS and 1 mM EDTA and neutrophils were enriched using EasySep™ Mouse Neutrophil Enrichment Kit (STEMCELL,19762) according to the manufacturer’s instruction.

### scRNA-seq and data analysis

#### Library construction and sequencing

Single-cell suspensions were prepared according to the protocol of Chromium Single Cell 3′ Solution (V3 chemistry). Reverse transcription and library preparation were performed using the 10× Genomics Single-Cell v3.0 kit following the 10× Genomics protocol. Single-cell libraries were submitted to 150 bp paired-end sequencing on the Illumina NavoSeq platform. Preprocessing of the data was done using the 10× Genomics Cell Ranger software version 5.0.0 in default mode. The 10× Genomics supplied reference data for the mm10 assembly and corresponding gene annotation was used for alignment and quantification.

#### Quality control

For cell filtering, cells outside the 5th and 95th percentile with respect to the number of genes detected and the number of unique molecular identifiers (UMIs) were discarded. Genes expressed fewer than three cells were filtered out. Cells with a percentage of mitochondrial genes higher than 10% were removed. Seurat R package (version 4.0.2) was used for downstream principal component analysis (PCA) and uniform manifold approximation and projection (UMAP) analysis [24].

#### Normalization, integration and dimension reduction

Functions in the Seurat package was used for the following analyses. Data were log normalized using the “*Log Normalize*” method, and the top 2,000 variable features were identified on a per sample basis. Samples were then anchored and integrated using Canonical Correlation Analysis (CCA) for batch correction to avoid the batch effect of sample identity which might disrupt the downstream analysis. It then computes mutual nearest neighbors (MNN) in the CCA subspace and serve as “anchors” to correct the data. After scaling the data, linear and non-linear dimension reduction was performed by PCA of variable features and UMAP analysis, respectively, using the top 15 principle components.

#### Clustering, annotation and marker identification

Clustering was calculated using the functions *FindNeighbors* and *FindClusters*. Cluster-specific features were then queried against a set of canonical cell type-specific markers from the literature. Cell types in clusters were defined using the following marker genes: B cells (*Cd79a*), T cells (*Cd3e*), NK cells (*Ncr1*), monocytes (*Cd68*), neutrophils (*Hdc*). Data were visualized using Seurat package functions, including *DimPlot*, *FeaturePlot*, *DotPlot*, *VlnPlot* and *DoHeatmap*.

#### Neutrophil-specific sub-clustering

Using the function *Seurat*::subset, neutrophils were reanalyzed in isolation. Neutrophil-specific analysis was completed as described above (from identifying a new set of top 2,000 variable features through clustering and marker identification). Analyses of Differentially expressed genes (DEGs) were performed to identify marker genes for cell clusters. The *FindAllMarkers* function (logfc.threshold = 0.25, adjusted P value < 0.05 using the Bonferroni correction) was used to determine unique and/or highly enriched DEGs in one cluster compared to all other clusters. Cluster-specific DEG gene IDs were converted to Ensembl IDs and then to Entrez IDs. For each individual cluster, Entrez IDs were analyzed using *clusterProfiler*::enrichGO, and GO terms were identified (adjusted P value < 0.05 using the Benjamini-Hochberg method, false discovery rate < 0.1).

#### Scoring of biological processes

Individual cells were scored for their expression of gene signatures representing certain biological functions. For all signatures, functional scores were defined as the average normalized expression of corresponding genes. The neutrophil granule, maturation, ROS and chemotaxis signatures were provided in Supplementary Table S1.

#### Cluster-specific differential gene expression and pathway analysis

Differential gene expression and pathway analysis were performed between two samples of cells on normalized gene expression values. Genes were identified as DEGs if they had an adjusted P value < 0.05 using the Bonferroni correction method and had a log_2_ fold change > 0.5. Gene Ontology analysis was performed by using the R package clusterProfiler [25]. All DEGs were converted to their Ensemble IDs and subsequently their Entrez IDs (as described above) prior to being separated into upregulated and downregulated lists with their accompanying log_2_ fold changes. Each list was then analyzed separately to determine upregulated and downregulated GO terms (as described above). Upregulated and downregulated DEGs as well as GO terms were compared across clusters and were visualized using *UpSetR* package. Lists of DEGs and pathways are provided in Supplementary Tables S2–S3.

### Flow cytometry

Female 7-month-old APP/PS1 and 5×FAD transgenic mice were anesthetized with 50 mg/kg sodium pentobarbital by intraperitoneal injection. Peripheral blood (600–800 µL) was collected by retro-orbital bleeding and diluted with 5 mL RBC lysis buffer (BD Biosciences) for 15 min at room temperature. 10 mL Hanks’ balanced salt solution (HBSS) were added to stop lysis followed by centrifugation for 10 min at 500 g. Next, cells were washed twice with 10 mL HBSS including 2 mM EDTA and 1% bovine serum albumin (BSA) and gently filtered through a 70 μm cell strainer before being resuspended in FACS buffer. All staining procedures were performed in FACS buffer. Before surface staining, cells were incubated with anti-CD16/CD32 (for Fc receptor blocking; 553140, BD Biosciences) for 15 min at 4 °C. The following antibodies were used: anti-CD45 (557659, BD Biosciences), anti-Ly6G (560559, BD Biosciences), anti-CCRL2 (564946, BD Biosciences), anti-CD11b (552850, BD Biosciences) and Fixable Viability Stain (564406, BD Biosciences). For surface staining, cells were incubated on ice for 30 min in the dark with the appropriate antibodies. Unbound antibodies were washed from cells with stain buffer and cells were resuspended in an appropriate volume of stain buffer for flow cytometric analysis. Flow cytometry data were acquired on the BD FACSAria III flow cytometer and analyzed with FlowJo software v10 (TreeStar).

### Immunohistochemistry

Female 7-month-old APP/PS1 mice were anesthetized with 50 mg/kg sodium pentobarbital by intraperitoneal injection and subjected to cardiac perfusion with ice-cold 0.1 M PBS followed by 4% paraformaldehyde. The brains were dissected and transferred to 4% paraformaldehyde for 24 h, and then to 30% sucrose solution until they were saturated. Then the brains were embedded in Tissue-Tek® O.C.T. Compound and frozen by liquid nitrogen. The brains were cut into coronal sections of 25 μm using a Leica CM 1950 cryostat. Brain sections were washed in PBS for 10 min, permeabilized with 0.3% Triton X-100 in PBS for 30 min, and blocked with PBS containing 0.2% Triton X-100, 10% FBS for 2 h at room temperature. The sections were then incubated with primary antibodies overnight at 4 °C, washed with PBS containing 0.1% Triton X-100, and incubated in fluorescence-conjugated secondary antibodies for 2 h at 37 °C. After washing with 0.1 M PBS, coverslips were counterstained with DAPI. The images were captured with a confocal laser microscope (LSM700, Carl Zeiss). The following primary antibodies were used: rat anti-Ly6G (BD biosciences, 551459, 1:200), rabbit anti-CCRL2 (Novus, NBP3-11982, 1:200), Alexa Fluor 594 goat anti-rat rabbit IgG (Invitrogen, A11007,1:500) and Alexa Fluor 488 goat anti- rabbit IgG (Invitrogen, A11034, 1:500).

### ALZDATA database for analysis

We used data from the ALZDATA one-stop database (http://www.alzdata.org), which collects high-throughput omics data and serves as an in-depth integrating system to integrate data of different levels. We downloaded the normalized data about differential *Ccrl2* expression of cross platform, including studies GSE12685, GSE36980, GSE48350, GSE5281, GSE53890, GSE66333, GSE15222, GSE29652, GSE37263, GSE26927 and GSE5281. Then we analyzed the normalized expression of *Ccrl2* in the entorhinal cortex (including GSE26927, GSE5281, GSE48350 and GSE26972), frontal cortex (including GSE48350, GSE53890, GSE36980 and GSE5281) and temporal cortex (including GSE5281,GSE36980 and GSE37263) of female AD patients and healthy humans aged from 54 to 106 years.

### Statistical analyses

For flow cytometry, data are expressed as mean ± s.e.m. Means between two groups were compared using two-tailed unpaired Student’s t-tests. *P* < 0.05 was considered statistically significant. Statistical analysis and graphics were made using GraphPad Prism (v.10.0).

## Results

### scRNA transcriptome profiling of neutrophils in APP/PS1 mice

**Fig. 1:**
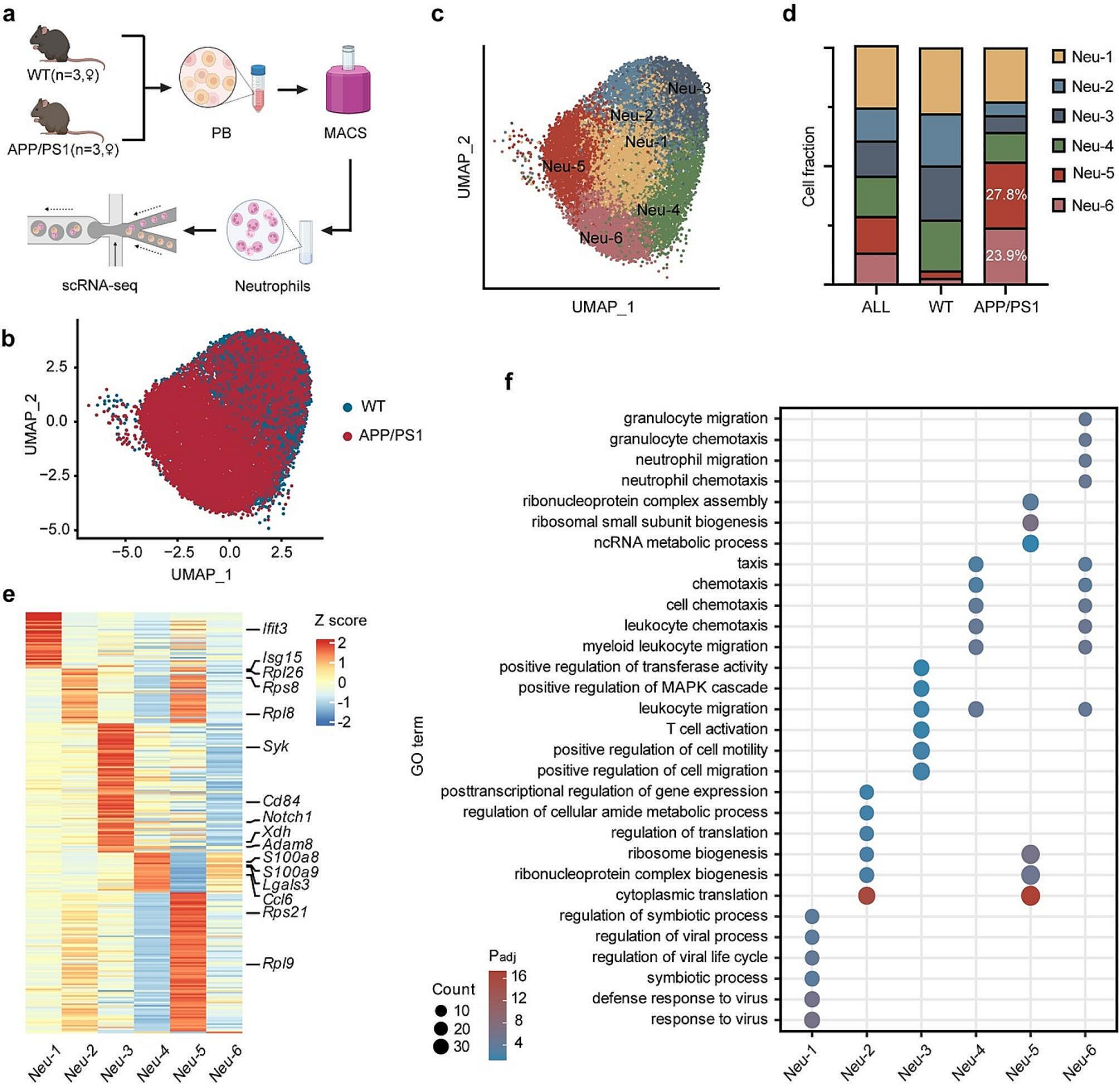
scRNA-seq analysis of peripheral blood neutrophils in APP/PS1 mice. (**a)** Schematic of the study design. (**b-c)** UMAP of 16,708 neutrophils, colored by sample (**b**) and cluster (**c**). (**d**) Proportions of the six neutrophil clusters in two samples. (**e**) Heatmap showing row-scaled expression of the differentially expressed genes per cluster for all neutrophils. (**f**) Functional annotation of neutrophil clusters using GO significantly enriched for their signature genes

To investigate the cell diversity and disease-related cellular changes in peripheral neutrophils in Alzheimer’s disease (AD), we performed 10× single-cell RNA sequencing (scRNA-seq). Neutrophils were isolated from blood of either wild-type (WT) or the APP/PS1 transgenic AD mice at 7 months of age, pooling cells from three mice (Fig. 1a). Following quality-control filtering, we retained 16,708 high-quality neutrophils, each with an average of 1,048 genes per cell profiled, resulting in a total of 17,527 mouse genes across all cells. We then performed robust batch correction on our data using a standard regression model (Methods section). Graph-based clustering was employed to identify cell clusters based on their unique gene expression profiles, and dimension reduction plots (Uniform Manifold Approximation and Projection, UMAP) were used for visualization. Analysis of canonical markers (*S100a9* and *Hdc*) confirmed the capture of blood neutrophils (Extended Data Fig. S1a, b), which constituted approximately 57% of the cells. Unsupervised cluster analysis partitioned the neutrophils into six clusters (Neu1-6) (Fig. 1b, c).

We assessed the distribution of each cluster by comparing the proportion of cells from APP/PS1 mice to those from WT mice. In UMAP space, cells from APP/PS1 mice segregated from those in WT mice, forming two distinct clusters, Neu-5 and Neu-6, while Neu-2, Neu-3 and Neu-4 primarily originated from WT mice (Fig. 1b, c). The proportion of Neu-1 was similar between the two samples. Neu-2, Neu-3 and Neu-4 were closely associated but more remote from Neu-5 and Neu-6 (Fig. 1d). There was substantial differential gene expression between the clusters (Fig. 1e).

In a gene ontology analysis of differentially expressed genes (DEGs), we noticed that the Neu-1 cluster expressed higher levels of interferon-stimulated genes (ISGs), such as *Ifit3* and *Slfn4*, compared to other clusters (Extended Data Fig. S1c). The Neu-2 cluster expressed more genes associated with ribosomal processes and translation initiation, such as *Rpl26* and *Rps8*. Notably, genes that were related to migration and chemotaxis were predominantly expressed in Neu-3, Neu-4, Neu-5 and Neu-6 clusters (Fig. 1f), suggesting that the motility of blood neutrophils in AD mice was affected.

### Assessing the characterization of neutrophil subclusters

**Fig. 2:**
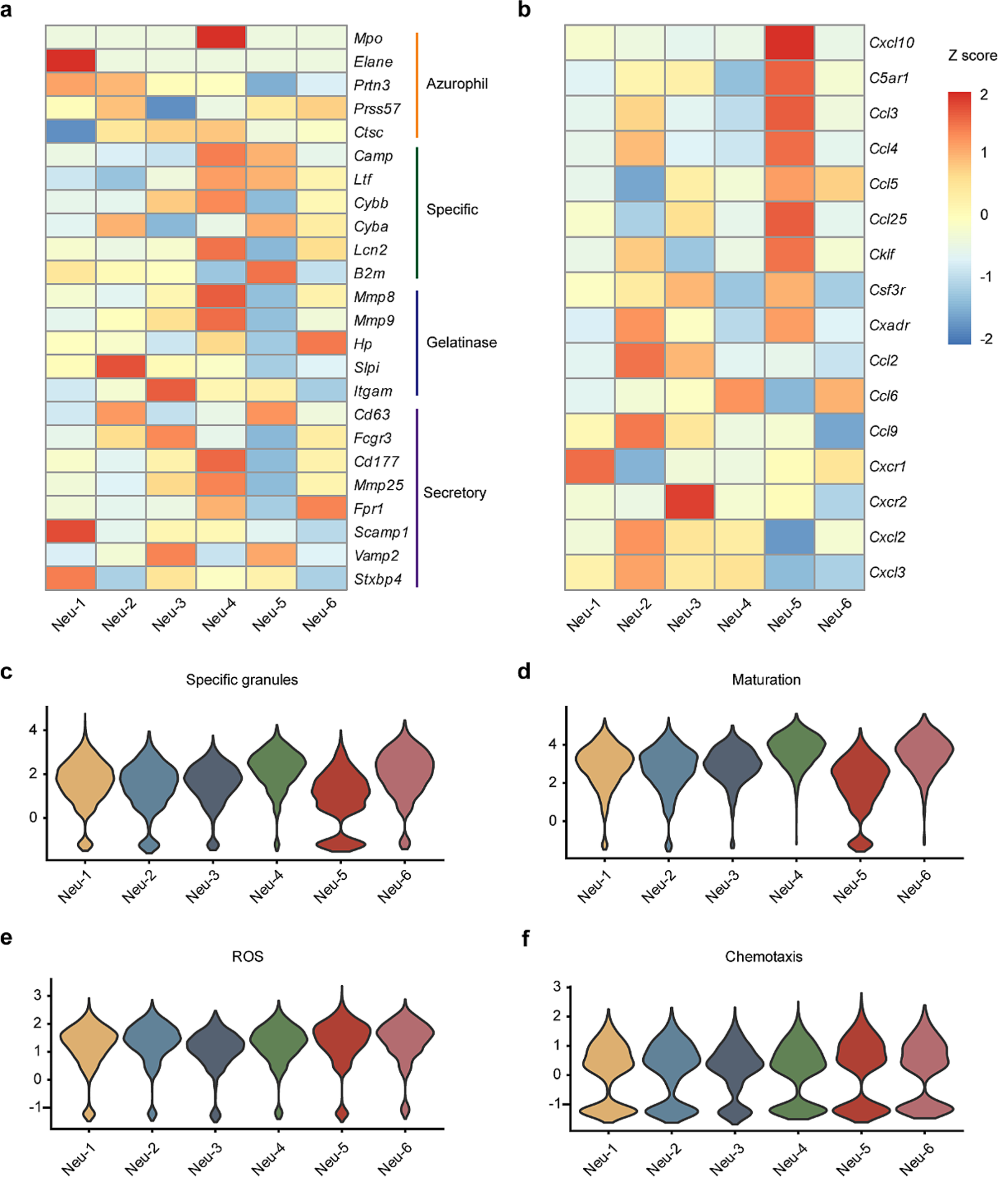
Functional characterization of neutrophil subclusters. (**a**) Heatmap showing the expression of neutrophil specific granule-related genes for all neutrophils. (**c**–**f**) Violin plots of specific granule score (**c**) maturation score (**d**), mitochondria-mediated ROS production score (reactive oxygen species biosynthetic process, GO:1,903,409) (**e**) and chemotaxis score (GO:0030593) (**f**) for each cluster. (**b**) Heatmap showing the expression of chemokine genes for all neutrophils

We next determined the characteristics of these clusters. The expression of granule genes in each cluster was evaluated. The Neu-1 cluster expressed higher levels of either azurophil or secretory granule genes, such as *Elane and Scamp1*, while the Neu-2 and Neu-3 clusters both displayed high expression of gelatinase granule genes. Moreover, Neu-4 showed high expression of all four different types of granules genes. However, the Neu-5 and Neu-6 clusters had lower expression of these granule genes, which was also illustrated by the granule score (Fig. 2a, c, Extended Data Fig. S2a–c). Furthermore, we analyzed the biological functions of these clusters. Compared to other clusters, the Neu-5 cluster showed a significantly lower maturation score, but higher ROS and chemotaxis scores (Fig. 2d–f), which indicated that more immature neutrophils with potentially higher activation and migration ability were present in the blood of AD mice. In addition, we also assessed the chemokines secreted by these clusters. The Neu-5 population obviously expressed higher levels of chemokines such as *Cx3cl1*, *Cxcl10*, *Ccl3*, *Ccl4* and *Ccl5* (Fig. 2b), which are responsible for the recruitment of T cells, natural killer cells and monocytes [26, 27].

### Cluster-specific transcriptome features

To examine the global transcriptomic changes in neutrophil subclusters, we compared the individual cluster transcriptome profiles between APP/PS1 and WT mice. The mean number of unique molecular identifiers (UMIs) and genes detected per cell in each cluster was comparable between the two samples. At the same sequencing depth, both the gene number and total UMIs increased in blood neutrophils of APP/PS1 mice, indicating elevated transcriptional activity during AD pathology (Extended Data Fig. S3a, b).

**Fig. 3:**
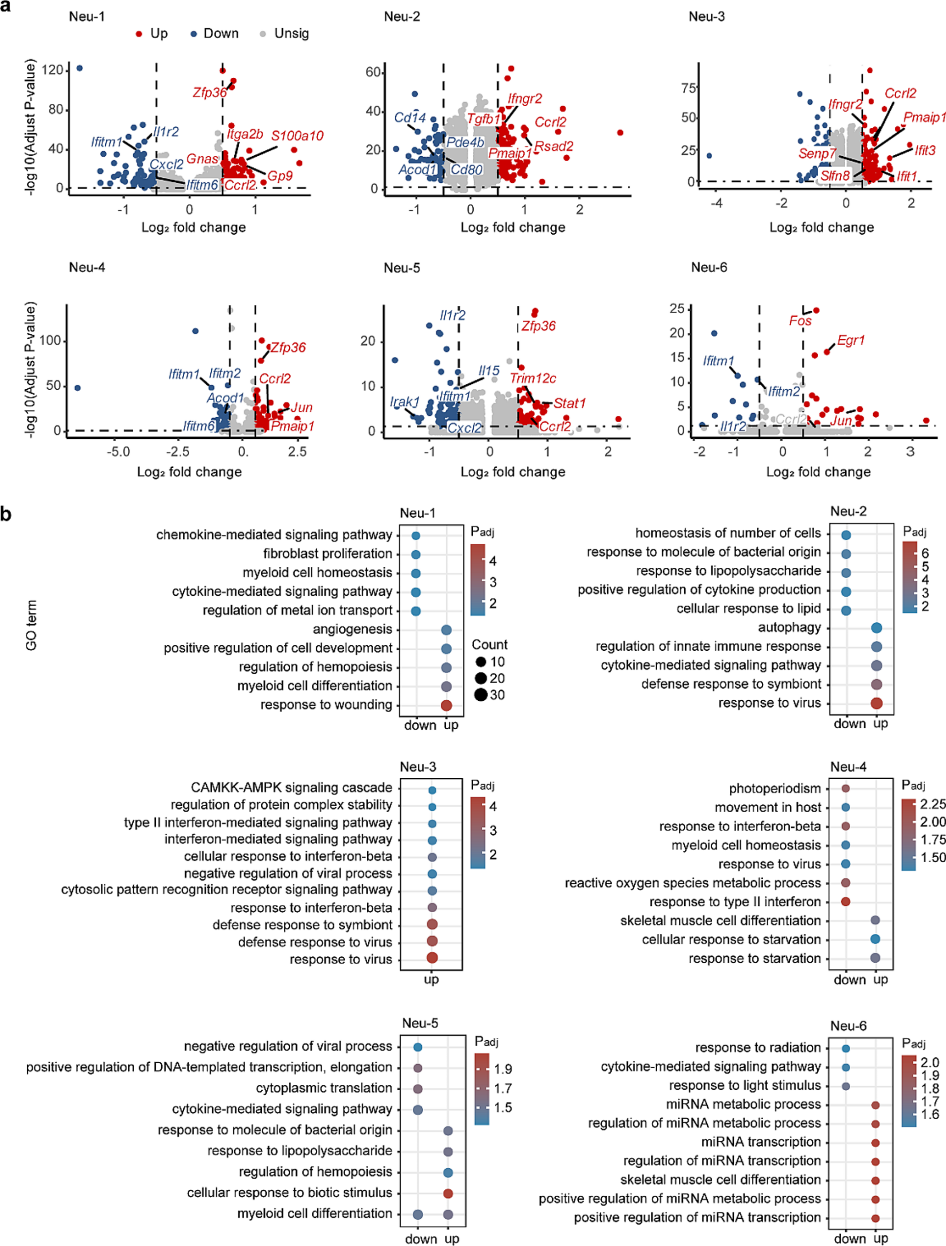
Cluster-specific changes in each neutrophil subpopulation in APP/PS1 mice. (**a**) Volcano plots displaying genes that are up- (red) or down-regulated (blue) in APP/PS1 mice for each cluster. Dashed lines denote fold change thresholds used when identifying DEGs. (**b**) GO analysis of DEGs in APP/PS1 mice for each cluster. Selected GO terms with Benjamini-Hochberg-corrected P-values < 0.05 (one-sided Fisher’s exact test) are shown

We identified a total of 2,190 DEGs (adjusted P value < 0.05) between APP/PS1 and WT mice, of which 171 DEGs were expressed in Neu-1, while 221 DEGs in Neu-2, 267 DEGs in Neu-3, 193 DEGs in Neu-4, 158 DEGs in Neu-5, and 30 DEGs in Neu-6 (Fig. 3a). Notably, only 12 DEGs were expressed in all six clusters (Extended Data Fig. S3b), suggesting that the transcriptomic features are cluster-specific.

The pathways associated with DEGs were further investigated. The upregulated DEGs were mainly involved in defense response to stimulus like virus and lipopolysaccharide in all these neutrophil clusters except for the Neu-6, which was associated with RNA transcription processes. Otherwise, the downregulated DEGs such as *Cd14* and *Il1r2*, were largely responsible for cytokine-mediated signaling pathways, particularly in the Neu-2, Neu-5 and Neu-6 clusters (Fig. 3b). Based on these findings, the increased Neu-5 and Neu-6 clusters in APP/PS1 mice were likely to decrease the response of cytokine-mediated inflammation.

### Annotating the clusters of AD-associated neutrophils

**Fig. 4:**
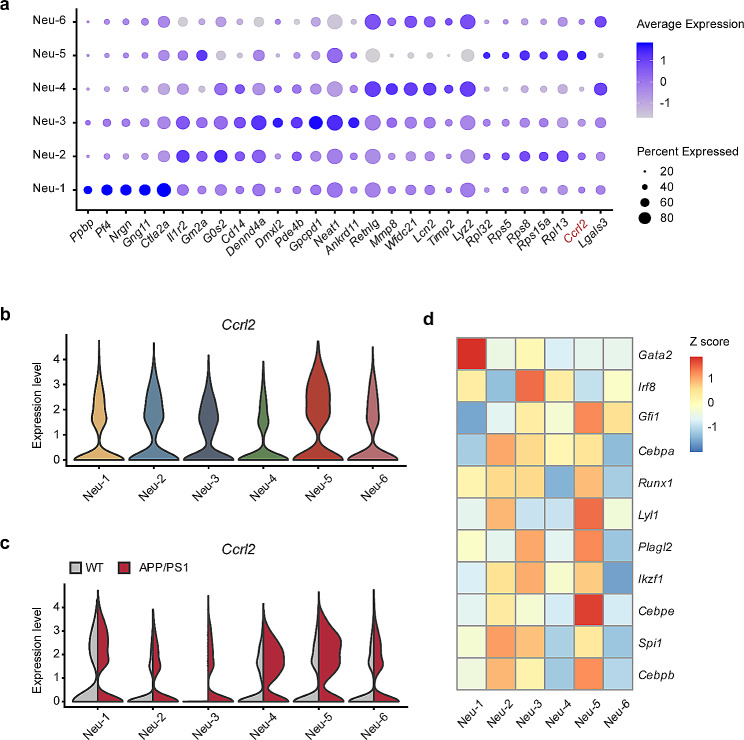
Novel molecular marker of AD-associated neutrophils. (**a**) Dot plot showing the scaled expression of signature genes for each cluster. (**b-c**) Comparisons of the expression level of *Ccrl2* for each cluster (**b**) and cross samples (**c**). (**d**) Heatmap of transcription factors known to regulate neutrophil granulopoiesis for each cluster

Based on the above data, we focused on the Neu-5 cluster, which demonstrated higher migration. Previous research has revealed a close correlation between neutrophils exhibiting a high migratory capacity and the pathological manifestations of AD [19]. To distinguish this cluster from others, we plotted the DEGs and found the *Ccrl2* gene was specifically enriched in the Neu-5 cluster. Further assessment of the *Ccrl2* gene expression among all clusters also validated the result (Fig. 4a-b), suggesting *Ccrl2* was a potential marker gene for the Neu-5 cluster. In addition, the *Ccrl2* gene in APP/PS1 mice was markedly upregulated across the six neutrophil clusters compared to WT mice (Fig. 4c). We next wondered which transcription factors drive the Neu-5 cluster. Noticeably, the *Cebpe* gene, which plays an essential role in specific and gelatinase granule formation, was highly expressed in the Neu-5 cluster (Fig. 4d).

### CCRL2 positive neutrophil populations were increased in AD model mice

**Fig. 5:**
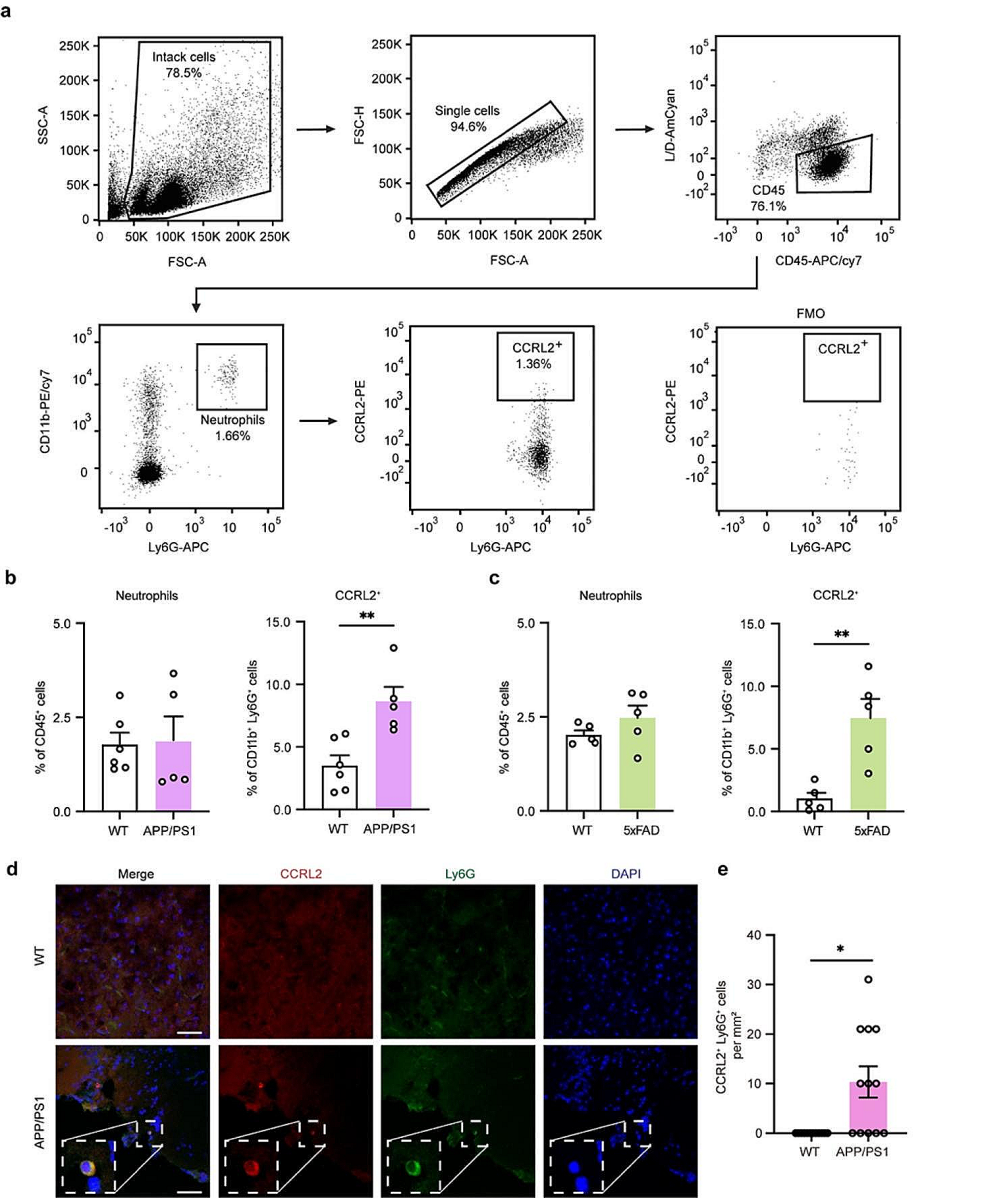
Analysis of CCRL2^+^ neutrophils by flow cytometry and immunohistochemistry. (**a**) Flow cytometry gating strategy for the identification of CCRL2 positive neutrophils. (**b-c**) Comparison of the population of neutrophils in CD45^+^ cells and CCRL2^+^ cells in CD11b^+^Ly6G^+^ cells from peripheral blood of female AD and wild-type mice (*n* = 5). Data shown as mean ± s.e.m.; and two-tailed unpaired *t*-test was used. (**d**) Images of CCRL2, Ly6G and DAPI in the cortex of 7-month-old female APP/PS1 mice. Scale bars, 50 μm. Boxed areas show the CCRL2 and Ly6G staining inset. (**e**) Quantification of the number of CCRL2^+^ Ly6G^+^ cells in female APP/PS1 (*n* = 12) and wild-type controls (*n* = 12) from d. Two-tailed, unpaired Student’s t-test was used and the CCRL2^+^ Ly6G^+^ cells were obviously higher (*p* = 0.0033) in the APP/PS1 mice compared to wild-type mice

We then examined the Neu-5 cluster by flow cytometric analysis in AD mice. We prepared blood neutrophils from APP/PS1 and 5×FAD mice, both of which are commonly used AD mouse models. The expression level of CCRL2 protein was quantified. CD45^+^CD11b^+^Ly6G^+^ cells were identified as neutrophils (Fig. 5a). The frequency of CCRL2^+^ neutrophils was significantly higher in both AD mice than WT mice. Meanwhile, the proportion of total neutrophils showed no difference between the mice (Fig. 5b-c). As reported, neutrophils invading into the brain promoted AD pathology [19] and CCRL2 protein plays a role in neutrophil migration [28]. Therefore, we sought to detect whether the increased CCRL2^+^ neutrophils in blood were also present in the brain parenchyma. By immunohistochemistry, we observed more CCRL2^+^ neutrophils in the brain of APP/PS1 mice (Fig. 5d-e).

### *Ccrl2* gene was upregulated in the temporal cortex of female AD patients

**Fig. 6:**
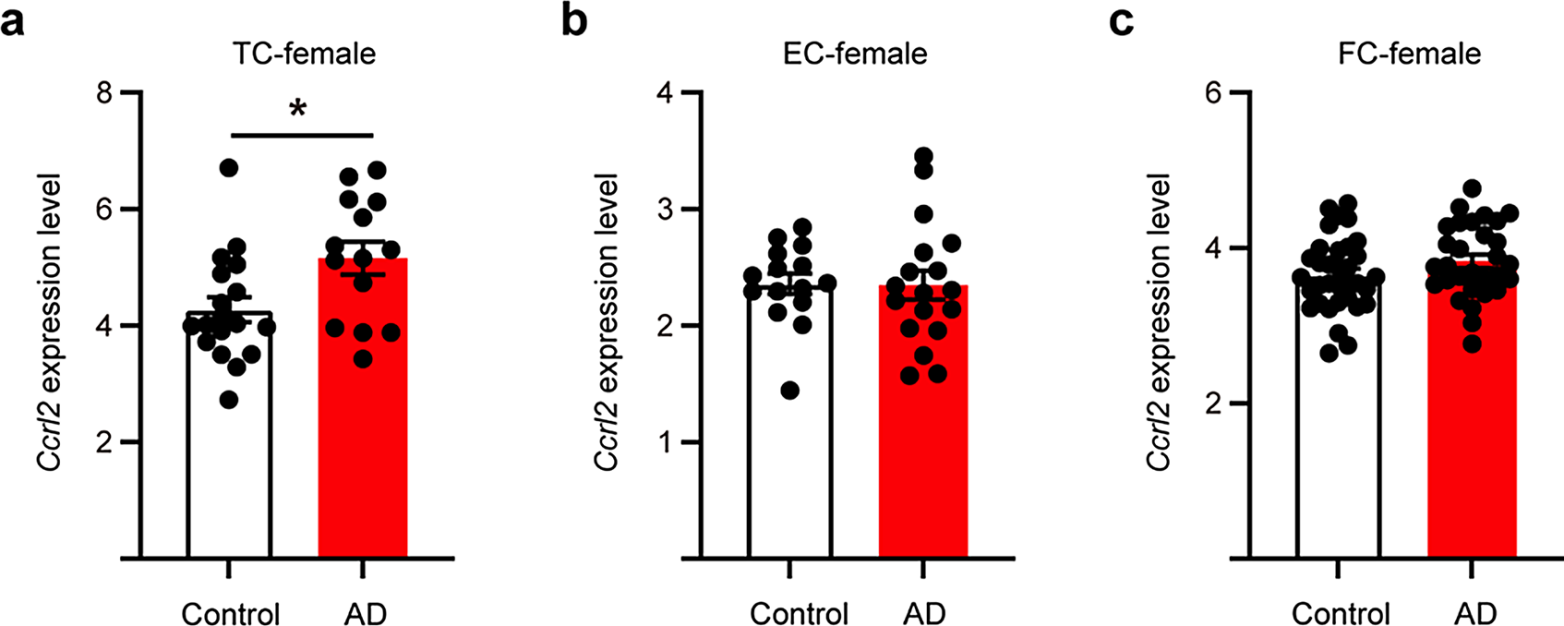
*Ccrl2* is specifically upregulated in female AD temporal cortex. (**a**) The expression level of *Ccrl2* in temporal cortex (TC) of female AD patients (*n* = 14) was significantly higher (*p* = 0.017) than healthy humans (*n* = 18). (**b**) The expression level of *Ccrl2* in entorhinal cortex (EC) of female AD patients (*n* = 18) and healthy humans (*n* = 15). (**c**) The expression level of *Ccrl2* in frontal cortex (FC) of female AD patients (*n* = 30) and healthy humans (*n* = 34). Data are shown as mean ± SEM and two-tailed, unpaired Student’s *t*-test was used. *p < 0.05

To explore the association of *Ccrl2* gene and AD, we used data from the ALZDATA database to assess the expression levels in different brain regions of female AD patients. Interestingly, the *Ccrl2* gene was obviously increased in the temporal cortex of female AD patients, while the frontal and entorhinal cortex showed no difference (Fig. 6a-c).

## Discussion

In this study, we identified six neutrophil subgroups in the blood of AD mice, among which the Neu-5 and Neu-6 clusters were obviously increased in AD mice. Following characterization, the Neu-5 cluster was closely associated with AD development, which exhibited fewer specific granules and a lower degree of mature. By DEGs analysis, we identified the Neu-5 cluster as *Ccrl2* gene positive, implying a higher tendency for chemotaxis, which was confirmed with flow cytometry and immunohistochemistry.

According to our data, the AD associated Neu-5 cluster comprised immature populations with few specific granules, indicating the cluster might not effectively defense against virus or bacterial infections. Recently, the scRNA-seq of neutrophils in different mouse organs suggested that immature neutrophils in the bone marrow were mobilized to the blood without undergoing full maturation [29]. In AD mice, it appears that more immature neutrophils were mobilized to the blood, thereby decreasing the number of functional neutrophils, as indicated by the Neu-4 cluster, which plays an indispensable role in the host defense system, characterized by its most advanced maturity level and granules. Another study also reported neutrophils in the peripheral blood of AD patients showed impaired phagocytosis, killing activity and secretion of inflammatory cytokines and chemokines [30].

Previous studies have reported neutrophils adhere to brain vessels and migrate into parenchyma in AD mouse models [19, 20], but little information was known about the invading neutrophils. In this work, we identified a CCRL2 positive neutrophil subgroup, which increased obviously in AD mice and showed higher ROS and chemotaxis scores. These data indicated that CCRL2 may promote neutrophil migration during AD pathology. CCRL2 is a nonsignaling seven-transmembrane domain receptor, which binds chemerin and promotes chemotaxis of leukocytes [31]. Prete et al. have reported that CCRL2-deficient mice showed defective neutrophil recruitment in inflamed joints, and therefore were protected from experimental models of inflammatory arthritis [28]. In human and mouse neutrophils, the expression of CCRL2 is upregulated by proinflammatory stimuli such as LPS or TNF-α alone or in combination with IFN-γ or GM-CSF [28, 32]. Numerous studies have validated the elevated TNF-α levels in the plasma of AD mouse models and patients [33–35]. Based on this, we speculated increased plasma TNF-α in AD mice upregulated CCRL2 expression in neutrophils, which favored migration and was associated with AD progression [19, 36]. Further experiments are needed to clarify the underlying mechanism.

Furthermore, we utilized the ALZDATA database to explore the correlation between the *Ccrl2* gene and AD among the Chinese population. Our analysis revealed that *Ccrl2* was particularly upregulated in the temporal cortex of females, but not in the frontal or entorhinal cortex (Fig. 6). Additionally, a previous study indicated a significant correlation between the expression level of *Ccrl2* and Aβ pathology in AD mouse models [37]. Therefore, it is possible that CCRL2, in addition to its role in peripheral blood, may also hold a crucial role in the pathology of AD within the brain. However, further evidence is required to substantiate this hypothesis.

In all, we identified the increased *Ccrl2*^+^ neutrophil subgroup in both peripheral blood and the brains of mice with AD. This finding implied a potential role for *Ccrl2*^+^ neutrophils in the pathogenesis of AD. Future research is needed to elucidate the specific function of CCRL2 protein in neutrophils and its contribution to AD progression. Such investigations could reveal a novel connection between circulating neutrophils and brain diseases.

## Conclusions

In summary, by scRNA-seq we stratified blood neutrophils from APP/PS1 mice into six distinct clusters and identified the notably increased CCRL2 positive neutrophil subgroup. This subset exhibited reduced expression of granule genes and a lower mature score. Remarkably, the CCRL2 positive neutrophils were also detected in the brains of AD mice, potentially indicating their involvement in the progression of AD. Our findings uncover a novel neutrophil subpopulation in the peripheral blood and brains of AD mice, offering a fresh perspective on the role of neutrophils in the pathogenesis of AD disease.

### Electronic supplementary material

Below is the link to the electronic supplementary material.

Supplementary Material 1

Supplementary Material 2

Supplementary Material 3

Supplementary Material 4

## Data Availability

Data supporting the findings of this study are available within the paper. All scRNA-seq data described in the paper have been deposited in the NCBI Gene Expression Omnibus (GEO) database and are accessible through the GEO SuperSeries accession number GSE255662.

## Code availability

All R codes used in this paper are available at https://github.com/Zhangxiaolin0/AD-neutrophil-scRNA-seq.

## Abbreviations

AD: Alzheimer’s Disease
LPS: Lipopolysaccharide
TNF-α: Tumor Necrosis Factor-α
IFN-γ: Interferon-γ
GM-CSF: Granulocyte-Macrophage Colony Stimulating Factor
scRNA-seq: Single-cell RNA Sequencing
DEGs: Differentially Expressed Genes
WT: Wild-Type
UMIs: Unique Molecular Identifiers
UMAP: Uniform Manifold Approximation and Projection
