# Supplementary material for: Single cell transcriptome analysis identified a unique neutrophil type associated with Alzheimer’s disease
Source: Immun Ageing. 2024 Jun 25;21:42. doi: 10.1186/s12979-024-00448-x (PMC11197360; doi:10.1186/s12979-024-00448-x)
Supplement: Supplementary file 4 — Supplementary Material 4 [file 12979_2024_448_MOESM4_ESM.docx]

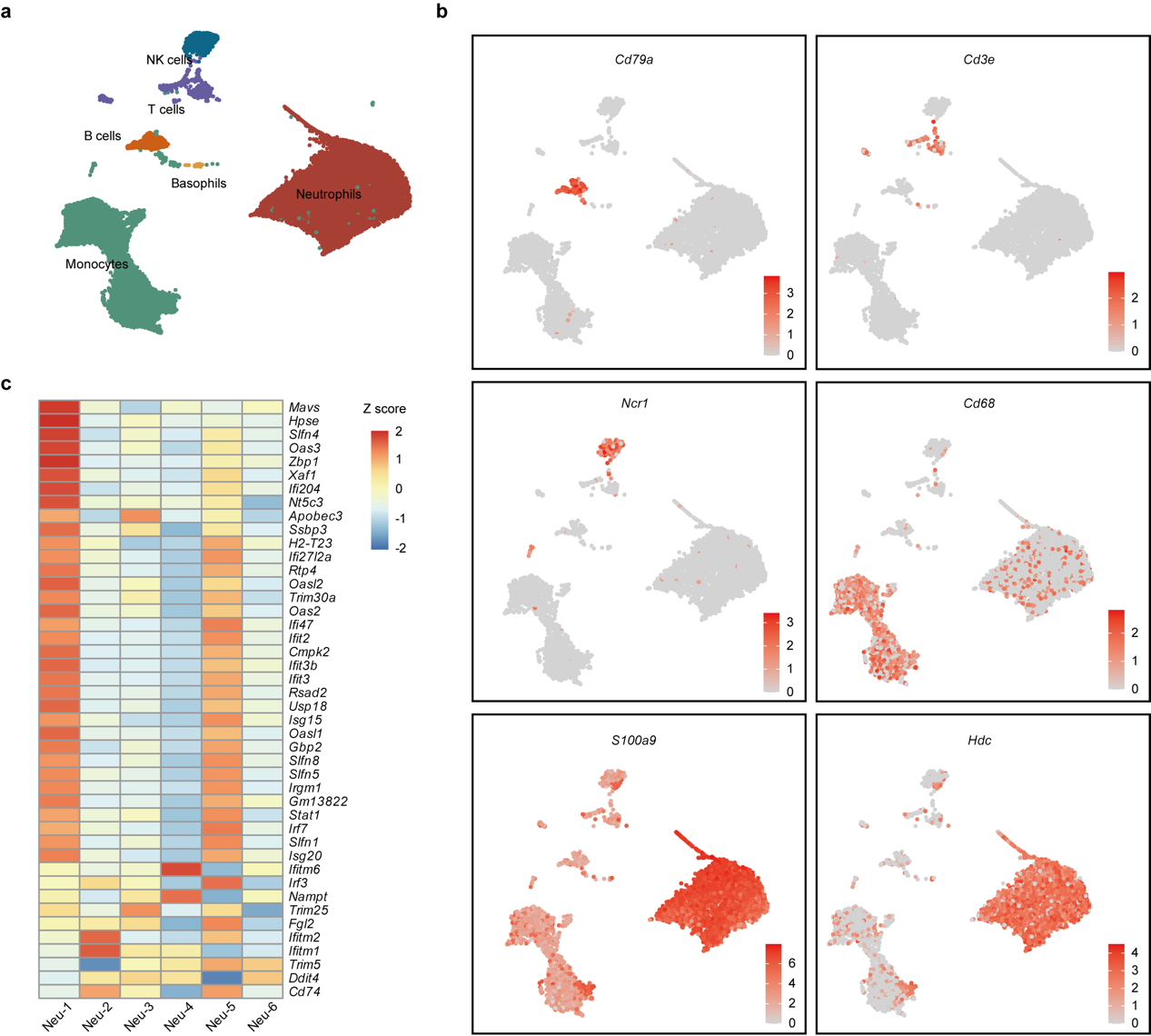


**Extended Data Fig. 1 scRNA-seq identifies peripheral blood immune cell types.**

**a**, UMAP of immune cell clusters colored by cell types from peripheral blood.

**b**, Expression of typical marker genes of immune cell types.

**c**, Heatmap showing the expression of ISGs for Neu-1 cluster.


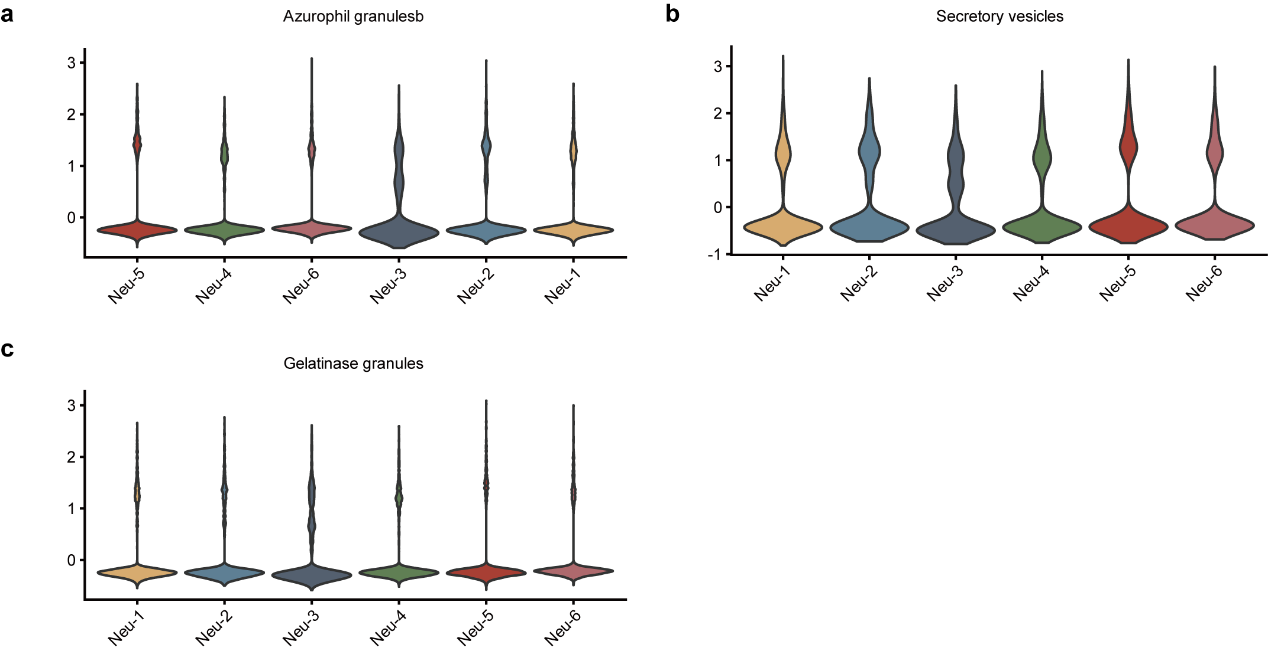


**Extended Data Fig. 2 Granule characterization of neutrophil subpopulations.**

**a-c**, Violin plots of azurophil score (**a**), secretory score (**b**) and gelatinase score (**c**) for each cluster.


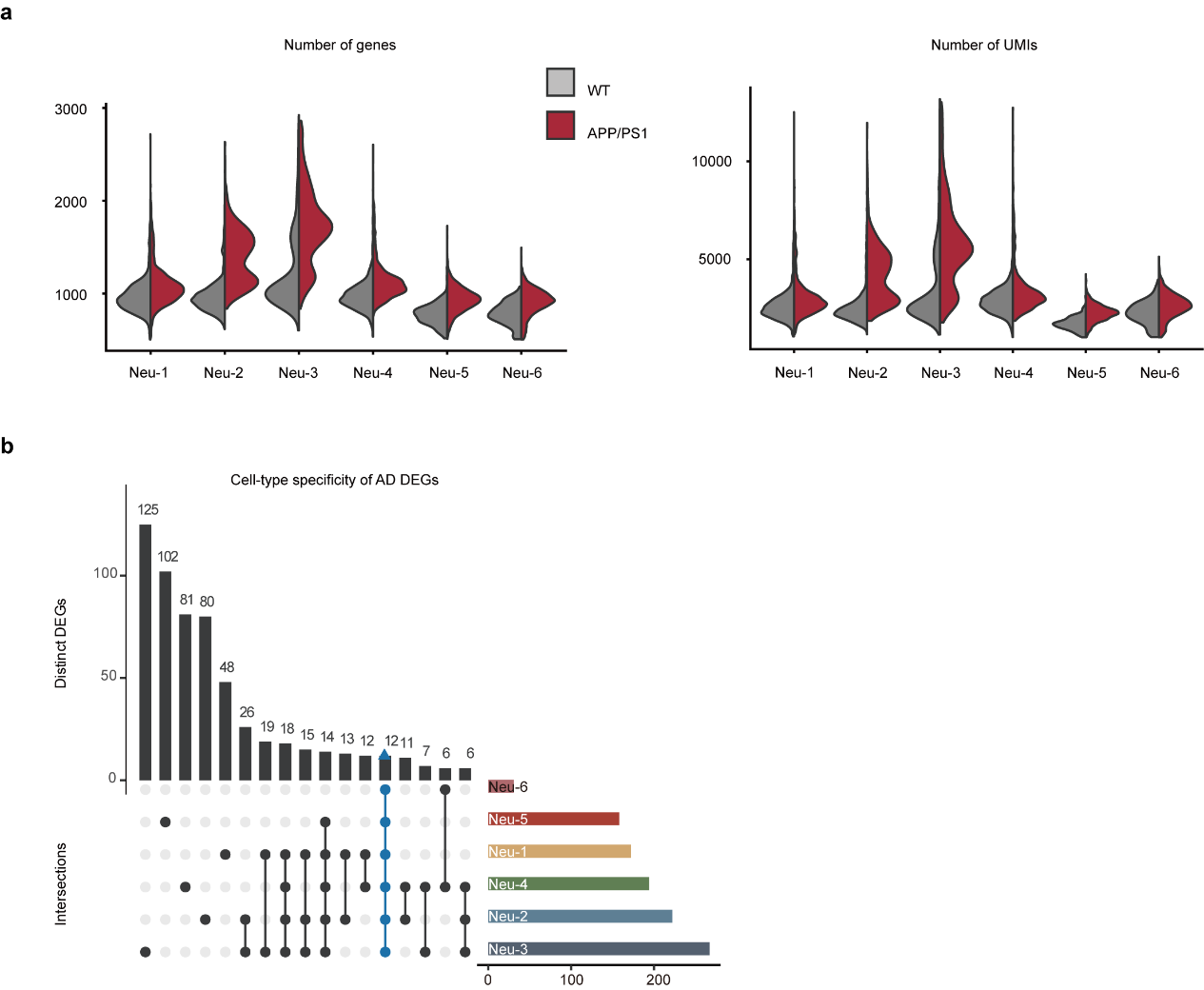


**Extended Data Fig. 3 Differentially gene activity in each neutrophil subpopulation in APP/PS1 mice.**

**a**, Comparisons of the number of genes and number of UMIs of all QC-passed cells in each cluster across samples.

**b**, Matrix layout for intersections of AD DEGs shared across and specific to each cluster. Circles in the matrix indicate sets that are part of the intersection.
